# Polymorphisms of the serotonin receptors genes in patients with bruxism: a systematic review

**DOI:** 10.1590/1678-7757-2021-0262

**Published:** 2022-01-07

**Authors:** Camilla Porto CAMPELLO, Sandra Lúcia Dantas MORAES, Belmiro Cavalcanti do Egito VASCONCELOS, Elker Lene Santos de LIMA, Eduardo Piza PELLIZZER, Cleidiel Aparecido Araújo LEMOS, Maria Tereza Cartaxo MUNIZ

**Affiliations:** 1 RENORBIO Brasil Rede Nordeste de Biotecnologia – RENORBIO, UFRPE/UPE, Programa de Pós-graduação em Biotecnologia, Recife, PE, Brasil.; UFRPE Programa de Pós-graduação em Biotecnologia Recife PE Brasil; 2 Universidade de Pernambuco Faculdade de Odontologia de Pernambuco Recife PE Brasil Universidade de Pernambuco – UPE, Faculdade de Odontologia de Pernambuco, Recife, PE, Brasil.; 3 Universidade de Pernambuco Faculdade de Ciências Médicas Programa de Pós-graduação em Ciências da Saúde Recife PE Brasil Universidade de Pernambuco – UPE, Faculdade de Ciências Médicas, Programa de Pós-graduação em Ciências da Saúde, Recife, PE, Brasil.; 4 Universidade Estadual Paulista Faculdade de Odontologia de Araçatuba Departamento de Materiais Odontológicos e Prótese Araçatuba SP Brasil Universidade Estadual Paulista – UNESP, Faculdade de Odontologia de Araçatuba, Departamento de Materiais Odontológicos e Prótese, Araçatuba, SP, Brasil.; 5 Universidade Federal de Juiz de Fora Departamento de Odontologia Governador Valadares MG Brasil Universidade Federal de Juiz de Fora – UFJF, Departamento de Odontologia, Governador Valadares, MG, Brasil.; 6 Universidade de Pernambuco Instituto de Ciências Biológicas Recife PE Brasil Universidade de Pernambuco – UPE, Instituto de Ciências Biológicas, Recife, PE, Brasil.

**Keywords:** Bruxism, Serotonin, Genetic polymorphism, Systematic review

## Abstract

This study aimed to investigate if SNP rs6313, SNP rs2770304, SNP rs4941573, and SNP rs1923884 of the 5-HT2A receptor gene and SNP rs6295 of the 5-HT1A receptor gene are associated with bruxism etiology.

**Methodology:**

This systematic review was registered in PROSPERO (CRD42018094561). A search was conducted for articles published in or before May 2021. To qualify for eligibility in this review, the studies had to be case-controls, cohort or cross-sectional. The inclusion criteria were the articles with a group of patients with bruxism and a control group in which the presence of these SNPs was evaluated. The exclusion criteria were the investigations of other polymorphisms, the studies that did not consider a control group for comparison, case reports, and reviews. The NOS and JBI were used to evaluate the methodological quality of studies.

**Results:**

We conducted this study with databases, such as Web of Science, Scopus, Embase, PubMed/MEDLINE, and ProQuest. We considered four studies eligible. A total of 672 participants were included,187 with sleep bruxism, 105 with awake bruxism, 89 with sleep and awake bruxism, and 291 controls. One study found a strong association between the SNPs rs6313, rs2770304 and rs4941573 of the 5-HT2A receptor gene and sleep bruxism. In one study, we considered the C allele of the SNP rs2770304 a risk factor for sleep bruxism. We found no significant results of other SNPs in sleep bruxers compared to controls. We found no positive association concerning the SNPs and groups of awake bruxism and sleep and awake bruxism.

**Conclusion:**

The different results regarding the SNPs in sleep bruxers could be explained by the genetic distinction between Chilean, Mexican, Japanese, and Polish population. More clinical trials and prospective studies must be conducted with larger sample size and in different ethnicities to confirm the results of this review.

## Introduction

Bruxism is defined as a repetitive jaw-muscle activity characterized by clenching or grinding of the teeth and/or by bracing or thrusting of the mandible.^1^ It has two different circadian manifestations: sleep bruxism occurring during sleep, characterized as rhythmic (phasic) or non-rhythmic (tonic); and awake bruxism, manifesting during wakefulness.^2^ Bruxism can cause damages, such as loss of tooth structure, cracked teeth, tooth hypersensitivity, pain in tooth, masticatory muscles, joint and face. It also affects 20% of the population and is more common in women than in men.^3^

The complete knowledge about the risk factors of bruxism is still unclear.^4^ Studies showed the involvement of genetic polymorphisms of *ACTN3*, *DRD2, ANKK1*, *5-HTT* and *COMT genes* with Bruxism risk.^5^^-^^7^ Serotonin (5-HT) is a neurotransmitter involved in the etiology of Bruxism.^8^ It is considerably the most important neurotransmitter that controls endogenous mechanisms of pain^9^ and it plays a role both in activation of the muscles^10^ and in the maintenance of chronic orofacial pain.^11^

The activity of 5-HT is mediated by 5-HT receptors.^12^ 5-HT1A and 5HT2A receptors are highly expressed in the prefrontal cortex.^13^*5-HT* receptors single nucleotide polymorphisms (SNP) have been associated with mood disorders,^14^ sleep quality^15^ and it is known to alter 5-HT mechanism of action.^14^*5-HT1A* is located on chromosome 5q11.2-q13 and presents the SNP rs6295 of the *5-HT1A* receptor gene.^16^

*5HT2A* is located on chromosome 13q14-21 and present in the first exon the T102C SNP rs *6313*.^17^ It has a base in nucleotide position 102, which can be thymine (T) or cytosine (C) and that determine three possible genotypes TT, TC, or CC.^18^*5-HT2A* also presents the SNPs rs2770304 in intron 2 of the gene,^19^ rs4941573 in intron 3 of the gene^20^ and rs1923884.^6^

Previous investigations showed that some *5-HT* receptors gene polymorphisms were positively associated with bruxism,^6^^,^^21^ nevertheless other studies have not identified the same results,^22^^,^^23^ demonstrating the lacking consensus about this topic. Furthermore, indicators for bruxism treatment are needed and a multidisciplinary approach could lead to therapeutic success.^24^ Therefore, this study aimed to investigate if T102C SNP rs6313, SNP rs2770304, SNP rs4941573 and SNP rs1923884 of the *5-HT2A* receptor gene and SNP rs6295 of the *5-HT1A* receptor gene, are associated with bruxism etiology. The research hypothesis was that SNPs present in *5-HT* receptors genes are involved in bruxism etiology.

## Methodology

### Registry protocol

This article followed the Preferred Reporting Items for Systematic Reviews and Meta-Analyses (PRISMA) checklist^25^ and it was registered in PROSPERO under the number CRD42018094561.

### Eligibility criteria

A specific question conducted this systematic review based on the “population, intervention/exposition, control, and outcome” (PICO) criteria. The question was: “Are SNPs of the *5-HT* receptors genes associated with bruxism etiology?” For this question the participants were composed by patients diagnosed with bruxism (sleep and awake); the exposure was the detection of the SNPs in individuals with bruxism compared to controls. The outcome was the SNPs of the *5-HT* receptors genes are involved in the etiology of bruxism.

To qualify for eligibility in this review, the studies had to be case-controls, cohort or cross-sectional. The inclusion criteria were the articles with a group of patients with awake or sleep bruxism, and a control group in which the presence of the SNPs of the *5-HT1A* and *5-HT2A* receptors genes was evaluated, regardless of the method since it does not modify the individual’s genetic code.

The exclusion criteria were the investigations of other polymorphisms, studies that did not consider a control group for comparison, case reports and reviews.

### Search methods

**Figure 1:**
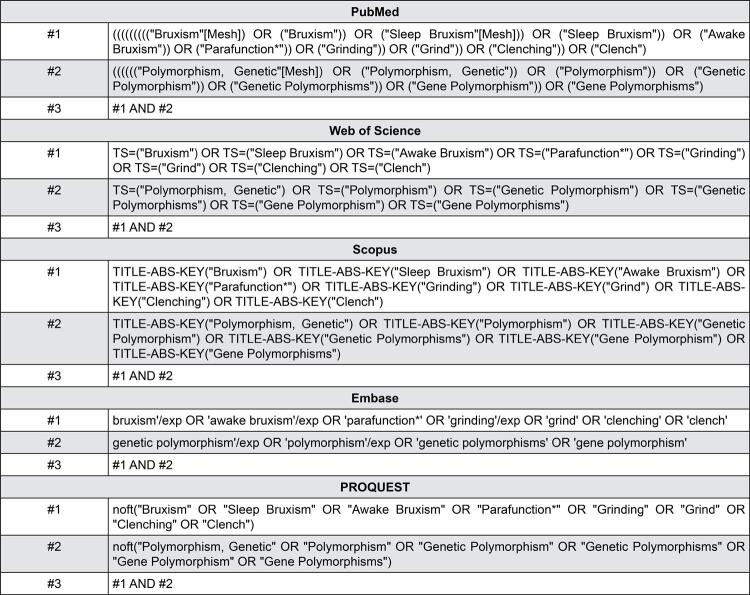
Search strategy in each electronic database

Two independent investigators (C.P.C and C.A.A.L) conducted a search in Web of Science, Scopus, Embase, and PubMed/MEDLINE databases (Figure 1), for articles published in or before May 2021. Moreover, the search in the grey literature for search of dissertations and theses were performed using the ProQuest databases, and the reference lists of eligible studies were hand-searched for additional reports. For the selection of studies, the Rayyan QCRI Software was used to perform these steps. The authors considered the exclusion of duplicate and reading the titles and summary of the articles. If there was not enough data in the title and summary, the whole article was acquired. Studies were excluded when they did not attend the inclusion criteria. All differences in selection process between the investigators were resolved by a third author (M.T.C.M.) to obtain a consensus through discussion.

### Data collection process

One author (C.P.C) collected the data from the articles and a second researcher (C.A.A.L) revised all the data extracted. A third investigator (B.C.E.V) examined all differences in choice between the investigators, thus there was a consensus. The variables collected were author, year, type of study, number of patients, number of healthy subjects, gender, age, and the presence of the SNPs of the *5-HT1A* and *5*-*HT2A* receptors genes.

### Quality assessment of included studies

Two authors (C.P.C. and C.A.A.L.) used the Newcastle-Ottawa Scale (NOS)^26^ to assess the risk of bias in the case-control and cohort studies. The NOS for case-controls studies analyzed the quality of studies based on the selection of study groups, their comparability and investigation of exposure, applying eight questions. The selection corresponds to four items: (1) Is the case definition adequate?; (2) Representativeness of the cases; (3) Selection of Controls; (4) Definition of Controls. Comparability consists to one item (1) Comparability of cases and controls based on the design or analysis. The Exposure category presents three items: (1) Analyses ascertainment of exposure; (2) Same method of ascertainment for cases and controls; and (3) non-Response rate. A maximum of one star can be attributed for each numbered item within the Selection and Exposure, whereas a maximum of two stars can be attributed for Comparability.

Along with the three categories of the NOS for cohort studies (selection, comparability, and outcome), eight items were analyzed. Selection presents four items: (1) Representativeness of the exposed cohort; (2) Selection of the non-exposed cohort; (3) Ascertainment of exposure; (4) Demonstration that outcome of interest was not present at the beginning of the study. Comparability corresponds to one item: (1) Comparability of cohorts based on the design or analysis. The outcome category is evaluated with three questions: (1) Assessment of outcome; (2) Was follow-up long enough for outcomes to occur; (3) Adequacy of follow up of cohorts. A maximum of one star can be attributed for each numbered item within the Selection and Outcome categories, whereas a maximum of two stars can be attributed to the Comparability.

Therefore, four stars could be attribute to the selection, two stars to the compatibility, and three stars to the exposure (case-controls studies)/outcome (Cohort studies). A maximum of nine stars can be attributed to a study, corresponding to the highest quality. Six stars or more are classified as low risk of bias, and five stars or less as high risk of bias.

Joanna Briggs Institute (JBI) Critical Appraisal Checklist for Analytical Cross-Sectional Studies was used to evaluate the methodological quality of these studies. The tool consists of eight questions that consider the evaluation of the inclusion criteria, details about the subjects and setting of study, valid and reliable exposure and outcomes measures, objective/standardized criteria for measuring the condition, identification of confounding factors, and the appropriate statistical analysis. The questions should have answers according to: Yes, No, Unclear, or Not/Applicable.^27^

### Summary Measures and additional analysis

Oporto, et al.^21^ (2016) and Cruz-Fierro, et al.^22^ (2018) did not provide the p-value of genotypic frequencies separately in groups of sleep bruxism, awake bruxism, and both. A study by Wieckiewicz, et al.^23^ (2020) included participants diagnosed with apnea; however, only patients diagnosed with sleep bruxism were considered in the analysis. Thus, BioEstat 5.3 software (Belém, Pará, Brazil) was used to identify if the polymorphisms of the *5-HT1A* and *5-HT2A* receptors genes influenced bruxism status in three separate groups: sleep bruxism, awake bruxism, and both. Univariate analyses using Chi-square tests at a 5% significance level were performed to verify if the genotypic frequencies of each SNP were associated to sleep bruxism, awake bruxism, and both compared with controls for the studies (Oporto, et al.^21^ (2016), Cruz-Fierro, et al.^22^ (2018) and Wieckiewicz, et al.^23^ (2020)). The other data were described as they are reported in the articles. The Kappa coefficient was estimated to find the inter-reader agreement at the time of inclusion of the articles of Web of Science, Scopus, Embase, PubMed/MEDLINE and ProQuest databases.

## Results

### Literature search

**Figure 2:**
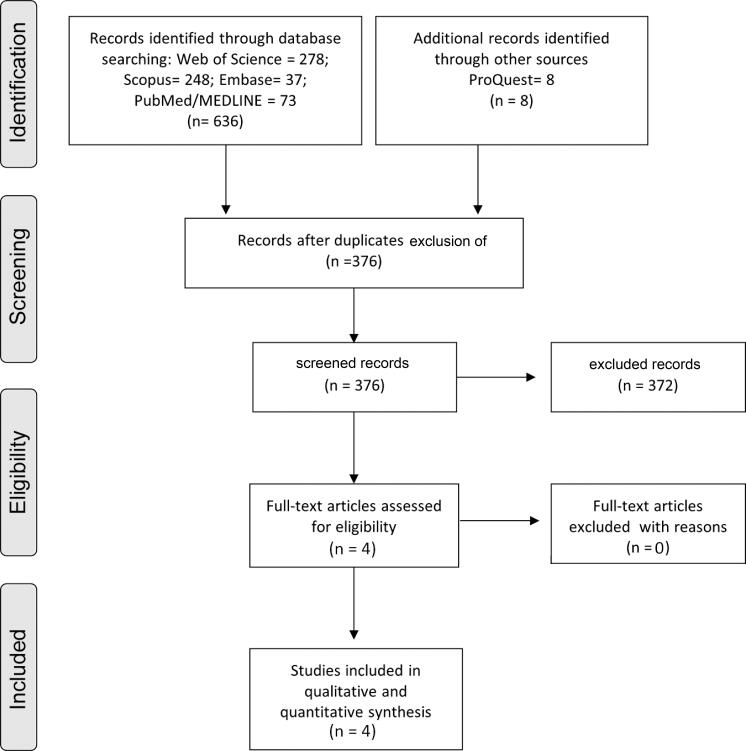
Prisma Flow Diagram

We presented the details of the search strategy in a flow diagram (Figure 2). It was conducted in databases, such as Web of Science (278), Scopus (248), Embase (37), PubMed/MEDLINE (75) and ProQuest (8) databases. A total of 376 articles remained after excluding duplicate references. After detailed review of the titles and abstracts and inclusion and exclusion criteria, we considered four studies eligible for this systematic review; Abe, et al.^6^ (2012); Cruz-Fierro, et al.^22^ (2018); Oporto, et al.^21^ (2016) and Wieckiewicz, et al.^23^ (2020). The kappa coefficient of inter-reader agreement showed a high level of accordance among the studies selected from the five databases (kappa value=0.90).

### Description of the studies

**Figure 3:**
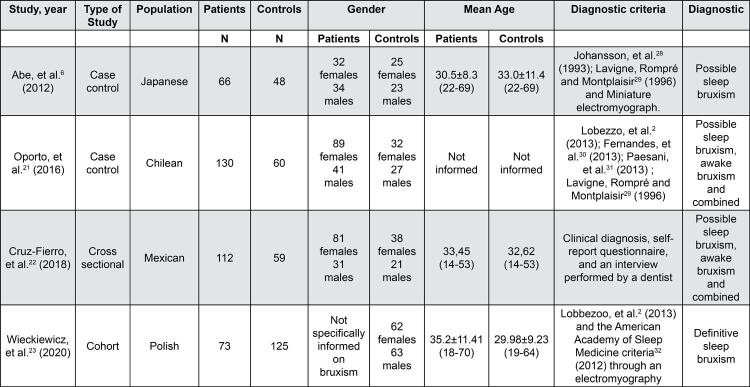
Characteristics of included studies

Figure 3 described details about the studies included in this systematic review. The included studies are case-controls; cohort; and cross-sectional with a group of cases and controls, that detected the presence of the T102C SNP rs6313, SNP rs2770304, SNP rs4941573 and SNP rs1923884 of the *5-HT2A* receptor gene and SNP rs6295 of the *5-HT1A* receptor gene.

### Participants of the studies

We included 672 participants in studies: 187 with sleep bruxism, 105 with awake bruxism, 89 with both awake and sleep forms, and 291 controls. In total, 157 were females, 106 were males and 73 were not informed, aged between 14 to 70 years old. The control group consisted of 157 females and 134 males, aged between 14 to 69 years old.

### Quality assessment and risk of bias of studies included

**Figure 4:**
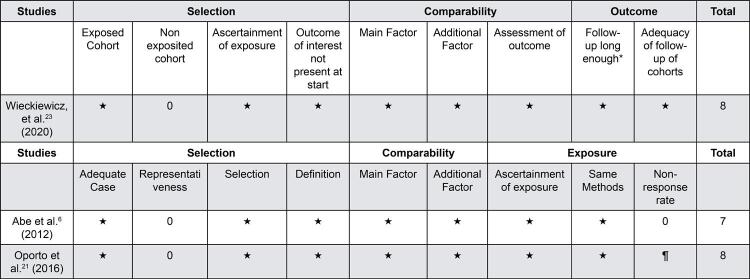
Assessment quality of Non-RCT included studies based on New Castle Ottawa

**Figure 5:**
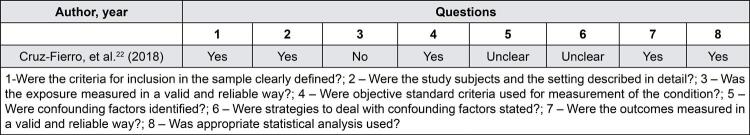
JBI Critical Appraisal Checklist for Analytical Cross-Sectional Studies

We analyzed the risk of bias through the NOS (Figure 4). We also associated the mostly absence of stars with a deficiency in terms of ascertainment of selection. According to JBI Critical Appraisal Checklist for Analytical Cross-Sectional Studies Cruz-Fierro, et al.^22^ (2018) presented five positive responses (Figure 5).

### The SNPs of the *5- HT1A* and *5-HT2A* receptors genes and sleep bruxism

**Figure 6:**
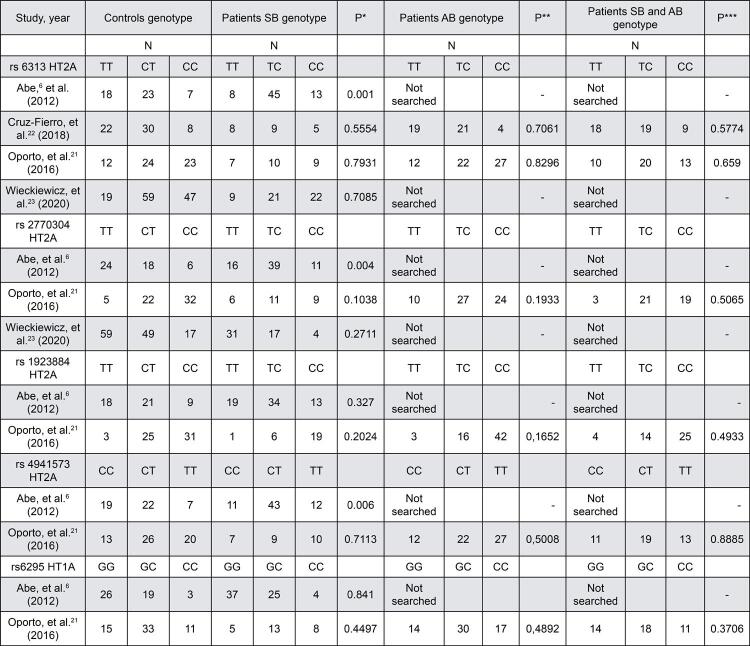
Genotypes of the SNPs of the 5- HT1A and 5-HT2A receptors genes in awake, sleep, or both bruxism

Along with four studies, we evaluated the presence of the SNPs of the *5-HT1A* and *5-HT2A* receptors genes in patients with sleep bruxism and controls (Figure 6). Three studies did not find a positive association between the T102C SNP of the 5-*HT2A* receptor gene and sleep bruxism (*p*=0.5554),^22^ (*p*=0.7931)^21^ and (*p*=0.2711).^23^

Three studies investigated the SNP rs2770304 of the *5-HT2A* receptor gene.^6^^,^^21^^,^^23^ According to Oporto, et al.^21^ (2016) the C allele of the SNP rs2770304 of the *5-HT2A* receptor gene was a risk factor for sleep bruxism development (OR=2.13; *p*=0.03 by Fisher test). In contrast, Wieckiewicz, et al.^23^ (2020) did not find a positive association between this SNP and sleep bruxism (*p*=0.2711).

Oporto, et al.^21^ (2016) illustrated an absence of association when compared patients with controls concerning the SNPs rs1923884 (*p*=0.2024) and rs4941573 of the *5-HT2A* receptor gene (*p*=0.7113), and the SNP rs6295 of the *5-HT1A* receptor gene (*p*=0.4497).

Abe, et al.^6^ (2012) showed different results compared to other studies. This investigation showed a strong association between the CC genotype of the T102C SNP of the 5-*HT2A* receptor gene and sleep bruxism (*p*=0.001). The C allele of this SNP was a risk factor for sleep bruxism (OR=4.250, *p*=0.004).^6^ We also found a meaningful association between the CC genotype of the SNP rs2770304 (*p*=0.004) and CC genotype of the SNP rs4941573 of the *5-HT2A* receptor gene (*p*=0.006) and sleep bruxism.^6^ We observed no significant difference in sleep bruxers compared to controls concerning the SNPs rs1923884 of the *5-HT2A* receptor gene (*p*=0.327) and the SNP rs6295 of the *5-HT1A* receptor gene (*p*=0.841).^6^

Different from the other investigations, this study excluded people without occlusal contact to the posterior teeth or with orofacial dysfunction, acute symptoms, systemic diseases, and everyone who use or used medication that modify the serotonergic system or sleep/wake regulation or treat disorders of movement.^6^ This research evaluated all participants (cases and controls) following Johansson, et al.^28^ (1993) and Lavigne, Rompré and Montplaisir.^29^ (1996) criteria. When this evaluation process could not identiﬁed sleep bruxers, they evaluated the participants using a miniature electromyograph.

Wieckiewicz, et al.^23^ (2020) evaluated cases with an electromyograph according to Lobbezoo, et al.^2^ criteria and the American Academy of Sleep Medicine criteria^32^, excluding subjects with malignant tumor, secondary bruxism associated with systemic diseases; who used medicines that can affect the nervous and muscular systems, and respiratory function; presented severe mental disorders and severe systemic diseases; severe mental retardation or Alzheimer’s disease; presented neurological disorders and/or neuropathic pain, respiratory insufficiency. Abe, et al.^6^ (2012) and Wieckiewicz, et al.^23^ (2020) performed a more complete assessment of patients compared to other articles. However, Wieckiewicz, et al.^23^ (2020) randomly selected healthy blood donors to compose the control group and did not perform bruxism evaluation of this group.

### The SNPs of the *5- HT1A* and *5-HT2A* receptors genes and awake bruxism

Two studies provided data from patients suffering from awake bruxism. Investigations did not find a positive association between the T102C SNP of the 5-*HT2A* receptor gene and awake bruxism (*p*=0.7061);^22^ (*p*=0.8296).^21^

Oporto, et al.^21^ (2016) was the only study that evaluated the SNPs rs2770304, SNP rs4941573, and SNP rs1923884 of the *5-HT2A* receptor gene and SNP rs6295 of the *5-HT1A* receptor gene in subjects with awake bruxism. We observed a lack of association when the presence of these SNPs was evaluated in patients compared to controls, SNP rs2770304 (*p*=0.1933), SNP rs4941573 (*p*=0.5008) and SNP rs1923884 of the *5-HT2A* receptor gene (*p*=0.1652) and SNP rs6295 of the *5-HT1A* receptor gene (*p*=0.4892).^21^

### The SNPs of the *5-HT1A* and *5-HT2A* receptors genes and sleep and awake bruxism

Two studies provided data from a group of patients with sleep and awake bruxism. They did not report a positive association between the T102C SNP of the *5-HT2A* receptor gene and individuals with awake and sleep bruxism (*p*=0.5754)^22^ and (*p*=0.659).^21^

Furthermore, only one study analyzed the frequency of other SNPs. We found no positive association between subjects with sleep and awake bruxism and the SNP rs2770304 (*p*=0.5065), SNP rs4941573 (*p*=0.8885) and SNP rs1923884 of the *5-HT2A* receptor gene (*p*=0.4933) and SNP rs6295 of the *5-HT1A* receptor gene (*p*=0.3706).^21^

## Discussion

In this systematic review, the research hypothesis that SNPs present in *5-HT* receptors genes are involved in bruxism etiology was accepted because the evaluated data from patients compared to controls suggest the implication of some SNPs of the *5-HT* receptors genes in bruxism etiology. According to Abe, et al.^6^ (2012) the CC genotype (polymorphic) of the SNP T102C of the *5-HT2A* receptor gene was positively associated with sleep bruxism etiology in the Japanese population (*p*=0.001). The C allele was considered a risk factor for sleep bruxism (OR=4.250, p=0.004).^6^ An investigation reported that the CC genotype of this SNP increased the risk of poor sleep quality compared to TT in a Chinese population (OR = 2.01, 95% CI: 1.25–3.23).^15^

On the other hand, other studies showed different results when sleep bruxers were compared with controls, in Chilean (*p*=0.7931),^21^ Mexican (*p*=0.5554)^22^ and Polish population (*p*=0.2711).^23^

Regarding the SNP rs2770304 of the *5-HT2A* receptor gene and sleep bruxism, Abe, et al.^6^ (2012) showed a meaningful association of the CC genotype of this SNP with sleep bruxism in Japanese population (*p*=0.004). Oporto, et al.^21^ (2016) reported that patients with the C allele of the SNP rs2770304 of the *5-HT2A* receptor gene were 2.13 times more likely to develop sleep bruxism compared to controls in Chilean population (OR=2.13; p=0.03 by Fisher test). Nevertheless, there was not a positive association between sleep bruxism and this SNP in Polish population (p=0.2711).^23^

We also observed controversial findings when the SNP rs4941573 of the *5-HT2A* receptor gene was analyzed in Japanese (*p*=0.006)^6^ and Chilean population (*p*=0.7113).^21^ The SNP rs1923884 of the *5-HT2A* receptor gene was not related with sleep bruxism in Japanese (*p*=0.327)^6^ and Chilean population (*p*=0.2024).^21^ Similarly, the SNP rs6295 of the *5-HT1A* receptor gene was not associated with sleep bruxism in both population (*p*=0.841)^6^ and (*p*=0.4497), respectively.^21^

The different results regarding the SNPs of the 5-*HT2A* receptor gene in sleep bruxers could be explained by the genetic distinction between the Chilean, Mexican, Japanese, and Polish populations.^6^^,^^21^^-^^23^

An absence of association between the T102C SNP of the *5-HT2A* receptor gene and awake bruxism was observed in Chilean (*p*=0.8296)^21^ and Mexican population (*p*=0.7061).^22^ Besides, we found no significant results concerning the awake bruxism and the SNP rs2770304 (*p*=0.1933), SNP rs4941573 (*p*=0.5008) and SNP rs1923884 of the *5-HT2A* receptor gene (*p*=0.1652) and SNP rs 6295 of the *5-HT1A* receptor gene (*p*=0.4892).^21^

We investigated subjects with sleep and awake bruxism and the presence of the T102C SNP of the *5-HT2A* receptor gene in Chilean and Mexican population, demonstrating no association in both nationalities (*p*=0.659)^21^ and (*p*=0.5754),^22^ respectively. We did not relate any of the SNPs with the group of patients with sleep and awake bruxism compared to controls.^21^

Sleep bruxism can cause pain in the masticatory muscles, limitation of jaw mobility, orofacial pain and headache in the temporal region.^33^ Most of the serotonin is present in the periphery and in the central nervous system, in the periphery system this neurotransmitter acts with other proinflammatory mediators to contribute to injury and inflammation causing pain.^34^ In deep craniofacial tissues, serotonin influences the induction of peripheral sensitization leading to the development of hyperalgesic nociceptive responses and contributes, essentially to the development and maintenance of chronic orofacial pain.^11^

In general, the studies presented a low risk of bias according to NOS because Abe, et al.^6^ (2012) scored seven stars. Oporto, et al.^21^ (2016) and Wieckiewicz, et al.^23^ (2020) had eight stars and Cruz-Fierro, et al.^22^ (2018) answered positively to five questions according to the JBI Critical Appraisal Checklist for Analytical Cross-Sectional Studies. However, the studies did not estimate the minimal sample size for the research. Cruz-Fierro, et al.^22^ (2018) reported a lack of accurate data on the prevalence of bruxism in Mexican population. This is the reason why this study was estimated in an infinitive population.

Other limitations of this systematic review were the study design, the low number of studies included, and the lack of standardization in diagnosis criteria, because the studies had different methodologies to diagnose bruxism (possible/probable bruxism or definitive bruxism) and it could influence the results. Furthermore, serotonin was also associated with anxiety, depression and neuroticism.^35^ Studies found the presence of these conditions in patients with bruxism^36^^-^^40^ and it also could affect the results. Abe, et al.^6^ (2012) and Cruz-Fierro, et al.^22^ (2018) evaluated personal traits. Only Cruz-Fierro, et al.^22^ (2018) study found a positive association between anxiety and neuroticism in sleep bruxers.

It is necessary to conduct more clinical trials and prospective studies with larger sample size, polysomnographic diagnosis of all participants and in different ethnicities to confirm the involvement of SNPs of the *5-HT1A* and *5-HT2A* receptors genes in bruxism etiology. These polymorphisms could be important biomarkers for stratification of patients.

Until now, we could not find a consensus in bruxism treatment.^4^ Diagnostic and management of pain are challenges in the orofacial region. 5HT receptors have been studying for a therapeutic planning for anxiety and depression.^41^ Future investigations can explore serotonin pathways in bruxism treatment.

## Conclusion

The analyzed data suggest the possible involvement of SNPs of the *5-HT* receptors genes in bruxism etiology. However, due to the limited evidence, further studies with larger sample size and different ethnicities should be considered to investigate the influence of polymorphisms in the involvement in awake and sleep bruxism etiology. Indicators for the development of effective new treatments could significantly improve the patients’ quality of life.
